# Three-dimensional tissue-engineered human skeletal muscle model of Pompe disease

**DOI:** 10.1038/s42003-021-02059-4

**Published:** 2021-05-05

**Authors:** Jason Wang, Chris J. Zhou, Alastair Khodabukus, Sabrina Tran, Sang-Oh Han, Aaron L. Carlson, Lauran Madden, Priya S. Kishnani, Dwight D. Koeberl, Nenad Bursac

**Affiliations:** 1 Department of Biomedical Engineering, Duke University, Durham, NC USA; 2 Division of Medical Genetics, Department of Pediatrics, Duke University School of Medicine, Durham, NC USA

**Keywords:** Musculoskeletal models, Metabolic disorders

## Abstract

In Pompe disease, the deficiency of the lysosomal enzyme acid alpha-glucosidase (GAA) causes skeletal and cardiac muscle weakness, respiratory failure, and premature death. While enzyme replacement therapy using recombinant human GAA (rhGAA) can significantly improve patient outcomes, detailed disease mechanisms and incomplete therapeutic effects require further studies. Here we report a three-dimensional primary human skeletal muscle (“myobundle”) model of infantile-onset Pompe disease (IOPD) that recapitulates hallmark pathological features including reduced GAA enzyme activity, elevated glycogen content and lysosome abundance, and increased sensitivity of muscle contractile function to metabolic stress. In vitro treatment of IOPD myobundles with rhGAA or adeno-associated virus (AAV)-mediated hGAA expression yields increased GAA activity and robust glycogen clearance, but no improvements in stress-induced functional deficits. We also apply RNA sequencing analysis to the quadriceps of untreated and AAV-treated GAA^−/−^ mice and wild-type controls to establish a Pompe disease-specific transcriptional signature and reveal novel disease pathways. The mouse-derived signature is enriched in the transcriptomic profile of IOPD vs. healthy myobundles and partially reversed by in vitro rhGAA treatment, further confirming the utility of the human myobundle model for studies of Pompe disease and therapy.

## Abstract (web summary)

Wang et al. describe the development of a 3D in vitro model of human skeletal muscle that recapitulates the pathophysiology of Pompe disease. They further define a Pompe disease-specific transcriptional signature, confirm its presence in the Pompe myobundles, and detect a partial reversal of the signature upon in vitro treatment of myobundles with rhGAA.

## Introduction

Pompe disease (glycogen storage disease type II, or acid maltase deficiency; OMIM 232300) is an autosomal-recessive disorder of metabolism caused by mutations in the lysosomal hydrolase, acid alpha-glucosidase gene (*GAA*). GAA normally breaks down lysosomal glycogen, and its dysfunction results in glycogen buildup in striated and smooth muscles, cardiac complications, progressive muscle atrophy, and respiratory failure^1^. The disease is categorized into two major types based on age of onset: infantile-onset Pompe disease (IOPD) and late-onset Pompe disease (LOPD) correlated with the level of residual enzyme activity^2,3^. Patients with the classic form of IOPD have little to no enzyme activity and develop hypertrophic cardiomyopathy and a progressive respiratory failure, leading to death within 1 year of age in absence of effective treatment. Patients with LOPD have greater GAA activity, but still have significant skeletal and smooth muscle involvement, difficulty walking, and progressively reduced respiratory function. Skeletal muscle weakness is common in both IOPD and LOPD^4,5^, driven by physical disruption of myofibrillar architecture related to enlarged and ruptured lysosomes, accumulated autophagosomes, and cellular debris^6,7^. However, the molecular mechanisms underlying the human disease are incompletely understood.

The current standard of care treatment for Pompe disease, enzyme replace therapy (ERT), consisting of frequent intravenous infusions of recombinant human GAA (rhGAA), reduces muscle glycogen and can improve muscle function and patient quality of life^8–11^. However, ERT has several limitations, including high cost^12^, attenuated efficacy due to neutralizing antibodies^13^, inefficient muscle targeting, and variable patient response^14^. Therefore, both improved understanding of the mechanisms driving the disease and new therapies with enhanced long-term efficacy are needed to improve patient outcomes.

Current in vivo studies of Pompe disease mainly utilize GAA^−/−^ mice^15^ that display a disease phenotype in between that of IOPD and LOPD, exhibiting glycogen accumulation but having delayed-onset of skeletal muscle weakness^15,16^ and only mild cardiac symptoms^16^. With almost 600 reported mutations in Pompe patients^17^ and the added complexity of modifier genes that can significantly alter disease severity even for the same mutation^18^, patient-specific human cell culture systems are necessary to model the wide spectrum of Pompe disease phenotypes. While two-dimensional (2D) cultures of patient-derived human myotubes have been utilized for this purpose^19,20^, they are not amenable to long-term culture, lack maturity relative to three-dimensional (3D) culture systems, and do not allow measurements of contractile function (twitch and tetanic contractions)^21^. Previously, we have developed a tissue-engineered 3D model of functional human skeletal muscle termed myobundles, using primary myoblasts^22,23^ or pluripotent stem cells^24^. These myobundles exhibit several features of native skeletal muscle (positive force-frequency relationship, length-tension relationship, mature dystrophin localization)^22,23^, adapt structurally and metabolically to electrical stimuli^22^, and demonstrate clinically relevant responses to tested drugs^23^. However, studies of Pompe disease in biomimetic functional 3D skeletal muscle culture systems have not yet been reported.

Here we sought to develop the first in vitro 3D model of human skeletal muscle with Pompe disease. We generated functional myobundles made from primary muscle cells derived from multiple patients with IOPD and healthy donors. Under standard culture conditions, IOPD myobundles exhibited GAA deficiency, elevated glycogen, and lysosomal enlargement, but displayed functional characteristics similar to those of healthy myobundles. Under stress conditions, including lysosomal perturbation, inhibition of glycogen phosphorylase, and glucose starvation, Pompe myobundles showed reduced tetanic force production, decreased fatigue resistance, and impaired glycogen mobilization, respectively. A 7-day treatment of IOPD myobundles with rhGAA or with adeno-associated virus (AAV)-mediated GAA expression significantly reduced glycogen accumulation, but did not rescue stress-induced functional deficits.

In addition, gene set enrichment analysis (GSEA) of RNA sequencing (RNA-seq) results indicated that IOPD myobundles exhibited transcriptomic changes characteristic of Pompe disease, including downregulation of gene sets involved in skeletal muscle contraction, increased endoplasmic reticulum stress, and reduced utilization of specific metabolic pathways. We also defined a muscle transcriptional signature of Pompe disease by comparing GAA^−/−^ and wild-type mice, confirmed the presence of this signature in IO Pompe myobundles, and detected a partial reversal of the signature upon in vitro treatment of myobundles with rhGAA.

## Results

### Structural and functional characterization of IO Pompe myobundles

**Fig. 1:**
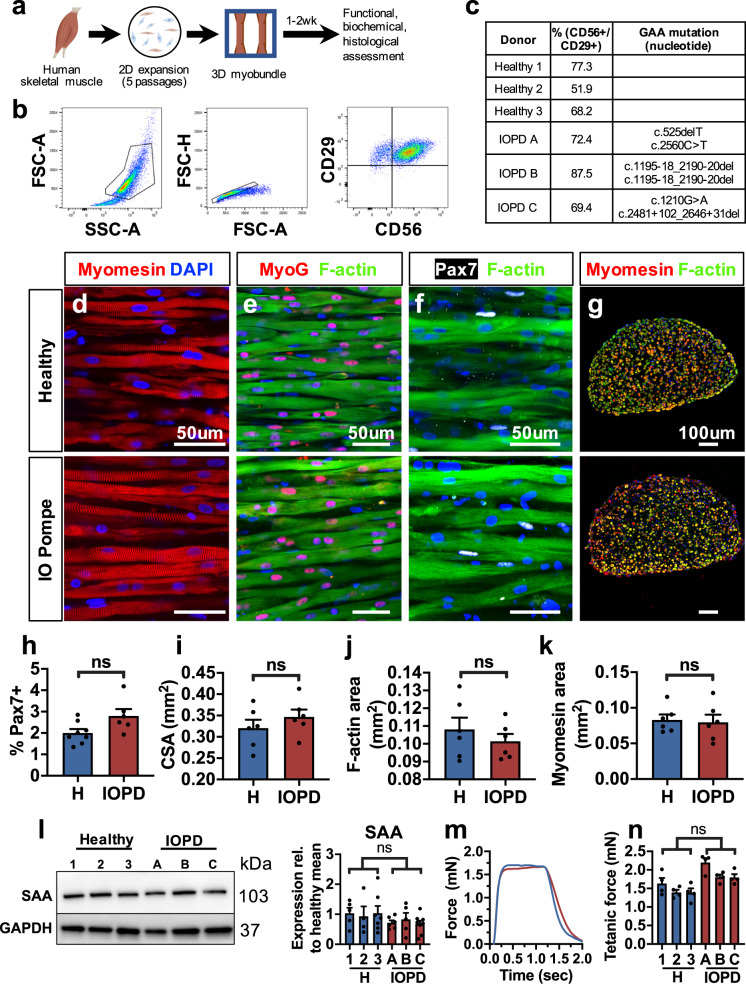
**Comparison of IOPD vs. healthy myobundle structure and function**. **a** Schematic of experimental flow from primary muscle cell isolation to myobundle fabrication and assessment. **b** Representative flow cytometry analysis of a muscle cell population expanded for five passages from IOPD patient biopsy, highlighting CD56+/CD29+ cell fraction. **c** Percentage of CD56+/CD29+ cells in expanded muscle cells and corresponding GAA mutations shown for each donor. **d**, **e** Representative longitudinal sections of healthy and IOPD myobundles differentiated in 3D culture for 2 weeks and stained for myomesin (**d**), myogenin and F-actin (**e**), and Pax7 and F-actin (**f**); nuclei are labeled with DAPI (blue). **g** Representative myobundle cross-sections stained for myomesin, F-actin, and DAPI. **h**–**k**, Quantification of immunostainings for (**h**) percentage of Pax7+ nuclei in total myobundle nuclei (*n* = 6–8), **i** total cross-sectional area (CSA) of myobundles (*n* = 6), **j** F-actin-positive CSA (*n* = 6), and **k** myomesin-positive CSA (*n* = 6). **l** Representative western blots and quantified protein expression (normalized to GAPDH) of sarcomeric α-actinin (SAA) in myobundles from three healthy (1, 2, 3) and three IOPD (A, B, C) donors (*n* = 4–8 myobundles per donor). **m**, **n** Representative 20 Hz tetanic force traces and **n** quantified force amplitudes in myobundles from healthy and IOPD donors (*n* = 4 myobundles per donor). Data: mean ± SEM. ns not significant.

To generate a 3D in vitro model of Pompe disease muscle, we have applied our method to generate functional human myobundles from primary muscle cells (Fig. 1a)^23^. Cells were isolated following enzymatic digestion and outgrowth culture from muscle biopsies of multiple healthy and IOPD donors and expanded for five passages. The myogenic cell fraction of expanded cells was quantified using flow cytometry for the myoblast surface antigens CD56^+^CD29^+^ (Fig. 1b, c)^25,26^ and found to be similar between healthy and Pompe disease cells (65.8 ± 7.4% and 76.4 ± 5.6% myoblasts, respectively). Passaged cells were embedded into a fibrin-based gel and differentiated for 2 weeks to form 3D tissue-engineered myobundles^22,23^. Similar to healthy myobundles, IOPD myobundles consisted of aligned, cross-striated, multinucleated myotubes that expressed the transcription factor myogenin, indicating terminal differentiation, while harboring a pool of Pax7+ satellite-like cells (Fig. 1d–g). The percentage of Pax7+ cells was similar between healthy and IOPD myobundles (Fig. 1h), consistent with previous histological assessment of human muscle biopises^27^. Morphological analysis revealed no difference in total, F-actin+, or myomesin+ cross-sectional areas (CSA) between healthy and IOPD myobundles (Fig. 1i–k), and, along with no difference in sarcomeric α-actinin (SAA) protein expression (Fig. 1l), revealed that IOPD satellite cells retained normal capacity for in vitro expansion, myofiber formation, and muscle differentiation.

We then assessed the force-generating capacity of myobundles from three healthy and three IOPD donors. Twitch responses of healthy and IOPD myobundles showed similar shapes and no significant differences in amplitude, time to peak twitch, or half relaxation time (Supplementary Fig. 1a–d). To compare the maximum force-generating capacities, tissues were stimulated at 20 Hz for 1 s to induce tetanus and, similar to twitch response, tetanic forces produced by healthy and IOPD myobundles were found to be comparable (Fig. 1m, n). Myobundles were also tested under a fatigue protocol in which tissues were held in tetanus for 30 s and their decline in generated contractile force was measured (Supplementary Fig. 1e)^22^. Greater resistance to fatigue is associated with reduced glycolysis and increased oxidative metabolism^28^. Healthy and IOPD myobundles displayed similar fatigue resistance based on two metrics: (1) drop in tension during stimulation and (2) impulse (area under the curve) normalized by peak force to cumulatively assess the force decline during fatigue testing (Supplementary Fig. 1f, g). Together, these results demonstrated that under normal culture conditions, 2-week old IOPD myobundles exhibited similar satellite cell abundance, muscle structure, and contractile properties to those of age-matched healthy myobundles.

### Reduced GAA activity and elevated glycogen in IOPD myobundles

**Fig. 2:**
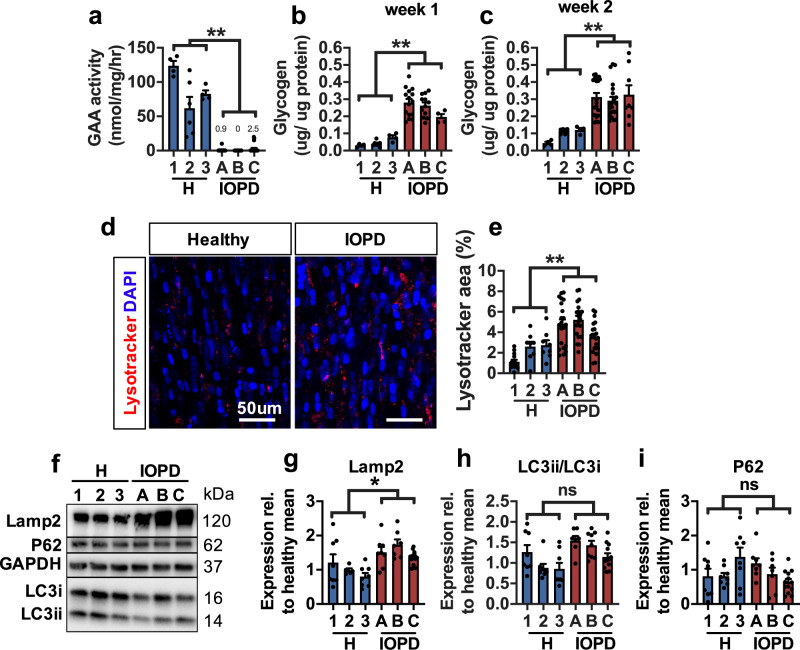
**Biochemical and morphological characteristics of IOPD myobundles**. **a** GAA activity in 3 healthy (1, 2, 3) and 3 IOPD (A, B, C) myobundles (*n* = 4–21 myobundles per donor). **b**, **c** Glycogen content after **b** 1 week (*n* = 4–12 myobundles per donor) and **c** 2 weeks (*n* = 4–21 myobundles per donor) of 3D differentiation. **d**, **e** Representative images of healthy and IOPD myobundles stained live with lysotracker and DAPI at 2 weeks of 3D differentiation (**d**) and corresponding quantification of percentage of myobundle area positive for lysotracker (**e**) (*n* = 8–21 myobundles per donor). **f**–**i** Representative western blots (**f**) and quantified protein expression of **g** Lamp2, **h** LC3-II/LC3-I ratio, and **i** p62/SQSTM1 (normalized to GAPDH) (*n* = 5–13 myobundles per donor). Data: mean ± SEM. *p < 0.05; **p < 0.01; ***p < 0.001; ns not significant.

In Pompe disease, impaired lysosomal GAA activity results in the buildup of glycogen in skeletal muscles, which prompted us to examine GAA activity and glycogen levels in healthy and IO Pompe myobundles (shown normalized by myobundle protein content). GAA activity in healthy myobundles was comparable to previously reported values from healthy human muscle biopsies^29^ and to other in vitro skeletal muscle models^19,20^ (Fig. 2a). Compared to these values, IOPD myobundles exhibited severely reduced GAA activity (Fig. 2a), consistent with human studies^29^. In addition, IOPD myobundles exhibited 4.9- and 3.1-fold greater glycogen content than healthy myobundles at week 1 and 2 of 3D differentiation, respectively (Fig. 2b, c). These results were unchanged when normalization was performed by cellular (approximated by GAPDH) instead of total myobundle protein (Supplementary Fig. 2). We also fabricated functional 3D human myobundles using myoblasts from two patients with late-onset Pompe disease (LOPD). The LOPD myobundles reduced GAA activity, yet not as low as IOPD myobundles (Supplementary Fig. 3a) and, unlike IOPD myobundles, did not exhibit elevated glycogen content (Supplementary Fig. 3b, c). This lack of phenotype in LOPD myobundles may be due to the longer disease progression timeline in LOPD, and is consistent with the observation that ~20% of patients with LOPD do not develop elevated glycogen^30,31^.

### Characterization of disease markers in IOPD myobundles

Glycogen buildup associated with IOPD causes lysosome swelling and dysfunction, resulting in impaired autophagy, autophagosome accumulation, and aggregation of proteins originally targeted for degradation^32,33^. To track lysosome abundance in myobundles, we stained 2-week-differentiated live myobundles with lysotracker, a pH-sensitive dye (Fig. 2d). IOPD myobundles showed 2.2-fold larger total area occupied by lysosomes compared to healthy myobundles (Fig. 2e) as well as augmented expression of the disease biomarker lysosome-associated membrane protein 2 (Lamp2, Fig. 2f, g), overall indicating increased lysosome abundance. Additionally, we assessed levels of microtubule-associated protein 2 light chain 3 (LC3-II), an autophagosome-specific protein that is converted from cytosolic LC3-I during autophagosome membrane formation and plays a key role in selecting cargo for degradation^34^. Impaired autophagy in Pompe disease results in an increased LC3-II/LC-I ratio and expression of p62/SQSTM1^35^, a multifunctional stress-inducible scaffold protein. After 2 weeks of differentiation, IOPD myobundles did not exhibit increased LC3-II/LC3-I ratio (Fig. 2f, h) or p62 expression (Fig. 2f, i) relative to healthy myobundles, consistent with findings in cultured myotubes derived from GAA^−/−^ mice^36^.

### Differential sensitivity of IOPD myobundles to lysosomal and metabolic stress

**Fig. 3:**
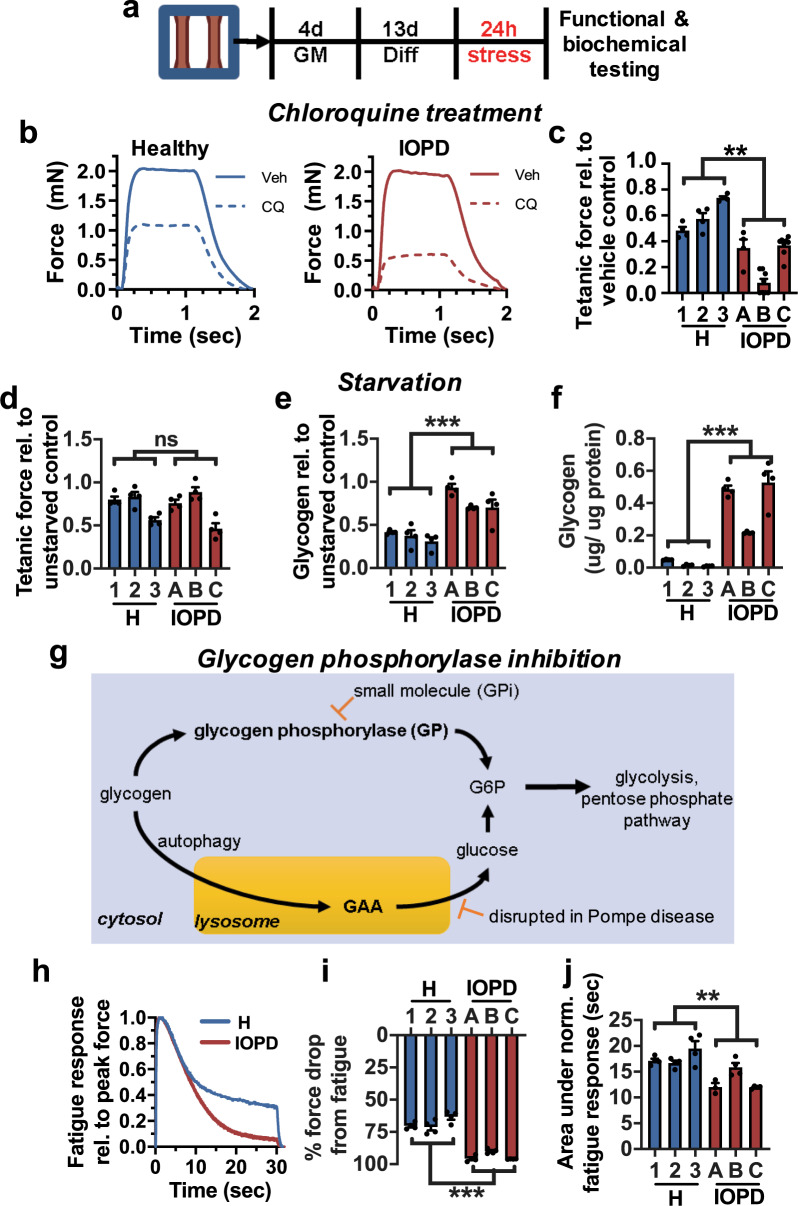
**Response of myobundles to metabolic stress**. **a** Schematic of experimental design: myobundles were cultured in growth media (GM) for 4 days and then differentiated for 13 days prior to 24 h of stress. **b** Representative force traces following 24 h of treatment with vehicle (Veh) or 5 µM chloroquine (CQ) in 3 healthy (1, 2, 3) and 3 IOPD (A, B, C). **c** Tetanic force after 24 h of treatment with 5 µM chloroquine shown relative to vehicle control (n = 4–8 myobundles per donor). **d** Tetanic force in myobundles after 24 h of glucose starvation relative to unstarved control (*n* = 4 myobundles per donor). **e** Glycogen content remaining after 24 h of glucose starvation (*n* = 4 myobundles per donor). **f** Fraction of glycogen remaining after 24 h of glucose starvation relative to unstarved control (*n* = 4 myobundles per donor). **g** Schematic of glycogen metabolism in skeletal muscle through lysosomal GAA and rate-limiting cytosolic glycogen phosphorylase (GP) pathways. **h** Representative force traces during fatigue test (20 Hz stimulation for 30 s) normalized to value of peak force, shown after 24 h of treatment with the GP inhibitor CP-91149 (10 µM). **i**, **j** Percent force decline at the end of fatigue stimulation (**i**, *n* = 4 myobundles per donor), and area under normalized force curve during fatigue stimulation (**j**, *n* = 4 myobundles per donor) after 24 h of treatment with the GP inhibitor. Data: mean ± SEM. *p < 0.05; **p < 0.01; ***p < 0.001; ns not significant.

Considering that under standard conditions IOPD myobundles showed no contractile deficit, we tested if they would reveal altered functional responses to stress in the form of chloroquine treatment, glucose starvation, or glycogen phosphorylase inhibition applied for 24 h at day 13 of differentiation (Fig. 3a). Chloroquine inhibits autophagosome-lysosome fusion and disrupts lysosomal membrane integrity^37,38^. When applied at 5 µM, it reduced force generation in the IOPD group to a greater extent than in the healthy group (Fig. 3b, c), reflecting the increased functional sensitivity of IOPD myobundles to lysosomal stress^39^. Glucose starvation for 24 h was applied to metabolically stress the myobundles, testing their ability to access stored glycogen. Although IOPD and healthy myobundles showed similar resulting tetanic forces (Fig. 3d), IOPD myobundles exhibited significantly impaired glycogen breakdown and mobilization compared to healthy myobundles as indicated by greater total remaining glycogen (Fig. 3e) and reduced glycogen depletion (Fig. 3f). Furthermore, in healthy native skeletal muscle, the cytoplasm rather than the lysosome is the primary site of glycogen storage^40,41^; thus, we hypothesized that the predominant reliance on the cytoplasmic glycogen degradation in myobundles may mask a pathological phenotype that depends on deficits in lysosomal glycogen degradation. We thus blocked cytoplasmic glycogenolysis for 24 h in healthy and IOPD myobundles by inhibiting the rate-limiting enzyme glycogen phosphorylase with the small molecule inhibitor (GPi) CP-91149 (Fig. 3g)^42,43^. While without the GPi, healthy and IOPD myobundles showed similar fatigue test responses (Supplementary Fig. 1e–g). After 10 μM GPi treatment, IOPD myobundles showed significantly reduced fatigue resistance compared to healthy myobundles (Fig. 3h–j), thus revealing another stress-induced functional deficit brought about by the underlying differences in overall glycogen metabolism.

### Recombinant GAA treatment of IOPD myobundles

**Fig. 4:**
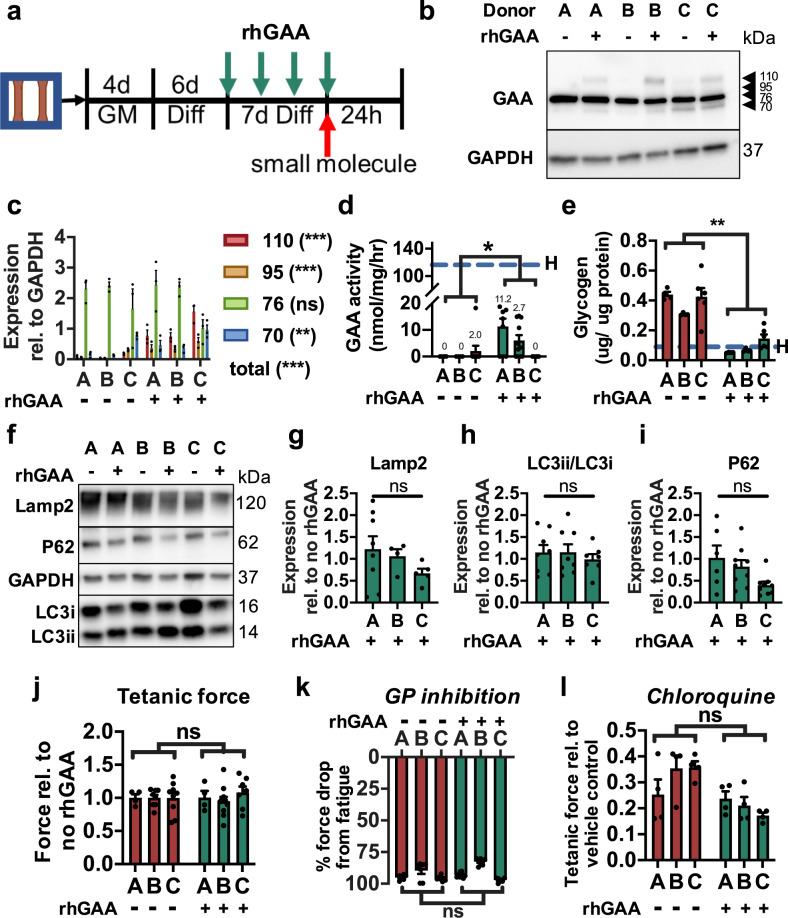
**Response of IOPD myobundles to rhGAA treatment**. **a** Schematics depicting protocol for rhGAA treatment of IO myobundles. **b**, **c**, Representative western blot of GAA isoforms in rhGAA treated (+) and untreated (−) myobundles from 3 IOPD patients (A, B, C) (**b**) and quantification of isoform expression relative to GAPDH (**c**, *n* = 3 myobundles per group). **d**, **e** GAA activity (**d**, *n* = 4–11 myobundles per group) and glycogen content (**e**, *n* = 4–6 myobundles per group) in IO myobundles after 1 week of rhGAA or no treatment. Dashed H-line denotes mean value across all healthy donors. **f**–**i** Representative western blot (**f**) and quantification of **g** Lamp2 expression, **h** LC3-II/LC3-I expression ratio, and **i** p62 expression (*n* = 4–9 myobundles per group) in IO myobundles after 1 week of rhGAA or no treatment. **j** Tetanic force of IO myobundles after 1 week of rhGAA treatment shown relative to no treatment (*n* = 4–11 myobundles per group). **k** Percent force decline at the end of fatigue stimulation after 24 h of GPi exposure following 1-week rhGAA treatment or no treatment (*n* = 6–8 myobundles per group). **l** Tetanic force after 24 h of chloroquine exposure following 1-week rhGAA treatment or no treatment, shown relative to vehicle control (*n* = 4 myobundles per group). Data: mean ± SEM. *p < 0.05; *p < 0.01; ns, not significant between rhGAA-treated and untreated myobundles.

The current standard of care for Pompe disease is ERT, wherein rhGAA enzyme is delivered biweekly to patients to reduce glycogen accumulation^44,45^. Successful delivery relies on endocytosis of the 110-kDa rhGAA enyzme and its transport to the lysosomes, during which several proteolytic processing steps cleave rhGAA to its mature and most catalytically active 76-kDa and 70-kDa isoforms^46,47^. To study the response of IOPD myobundles to ERT, we provided rhGAA at 2 µg/mL in fresh culture media every two days between differentiation days 7–14 (Fig. 4a). In examining the distribution of GAA isoforms by western blot, we found that rhGAA treatment resulted in successful uptake of the enzyme as indicated by greater levels of the 110-, 95-, and 70-kDa GAA isoforms but no significant increase in the 76-kDa isoform (Fig. 4b, c). This lack of increase in the 76-kDa band may be due to the already high expression of endogenous (dysfunctional) 76-kDa GAA in untreated myobundles, with a possibility that a fraction of the band was still contributed by rhGAA-derived (functional) 76-kDa isoform. Notably, rhGAA treatment resulted in a disproportionate increase in the immature 110-kDa isoform (11.7-fold versus no treatment) relative to the increase in the mature 70-kDa isoform (1.7-fold), possibly due to impaired trafficking of the enzyme observed in more advanced stages of the disease^48,49^. In comparison, rhGAA treatment of healthy myobundles resulted in neither disproportionate nor individual increases in any of the GAA isoforms (Supplementary Fig. 4a, b), suggesting that unknown feedback mechanisms may regulate homeostatic levels of GAA protein. Importantly, rhGAA treatment of IOPD myobundles significantly increased GAA enzyme activity, albeit below healthy levels (Fig. 4d), and reduced glycogen content by 77%, near levels measured in healthy myobundles (Fig. 4e). This 1-week rhGAA treatment was insufficient to significantly reduce Lamp2 expression (Fig. 4f, g), the LC3-II/LC3-I ratio (Fig. 4f, h), or p62 expression (Fig. 4f, i). rhGAA treatment also did not improve tetanic force generation (Fig. 4j) under standard culture conditions, attenuate GPi-induced decline in fatigue (Fig. 4k), or reduce chloroquine-induced loss of tetanic force (Fig. 4l).

### Effect of AAV-mediated GAA gene therapy on IOPD myobundles

Gene therapy is a promising single-treatment approach to restore robust GAA expression to diseased tissue^50^. Overexpression of GAA via intramuscular injection of recombinant (r) AAV vectors increases tissue GAA activity and reduces muscle glycogen in GAA^−/−^ mice^51^. To explore the effect of AAV gene therapy in our in vitro model, healthy and IOPD myobundles were transduced during myobundle fabrication with rAAV9 vectors encoding either hGAA^51^ or eGFP (control) driven by the muscle-specific regulatory cassette MHCK7^52^. After 2 weeks of 3D differentiation, successful myobundle transduction was confirmed by observing strong eGFP fluorescence in immunostained muscle fibers (Supplementary Fig. 5a). The rAAV9-MHCK7hGAA transduced IOPD myobundles showed both robust increase in GAA activity (Supplementary Fig. 5b) and a ~75% decrease in glycogen content (Supplementary Fig. 5c), reaching levels measured in healthy myobundles. Nevertheless, consistent with the rhGAA supplementation, vector treatment did not improve tetanic force production under standard conditions (Supplementary Fig. 5d) or reduce muscle fatigue and force loss in GPi-induced (Supplementary Fig. 5e) and chloroquine-induced (Supplementary Fig. 5f) stress conditions, respectively.

### Transcriptomic analysis of GAA^−/−^ (KO) mice and identification of a Pompe disease signature

To better understand the molecular signature underlying the Pompe disease phenotype and response to therapy, RNA sequencing (RNA-Seq) analysis was performed on the quadriceps of 6-month-old wild-type (WT) and GAA^−/−^ (KO) mice. As expected, KO mice showed lower GAA activity and greater glycogen accumulation than WT mice (Supplementary Fig. 6a, b). Transcriptomic analysis revealed 2244 genes that were significantly differentially expressed (|log_2_(fold-change)| ≥ 1, *p*_adj_ < 0.05) between WT and KO mice. Gene set enrichment analysis (GSEA) using the gene ontology (GO) annotation to investigate the significant functional categories describing coordinated gene expression revealed both expected and novel descriptors of Pompe disease (*q* < 10^−3^), including reduced protein translation (GO:0006414, GO:0006415), impaired mitochondrial function and oxidative metabolism (GO: 0006119), reduced ATP biosynthesis (GO:0006754), and increased cAMP signaling (GO:0046058) (Supplementary Fig. 6c), some of which are consistent with previous studies in GAA^−/−^ mice^4,39,53,54^. From the RNA-Seq data set, we also generated a Pompe disease signature that consisted of the top 50% of altered genes with |log_2_FC| ≥ 2 and *p*_adj_ < 0.05, of which 243 were downregulated and 39 were upregulated in GAA^−/−^ vs. WT mice (Supplementary Data 1a, b). Using the Reactome database (https://reactome.org/), constituent genes in each set were confirmed to span multiple categories consistent with Pompe disease phenotype, including muscle contraction, autophagy, amino acid and lipid metabolism, extracellular matrix organization, and protein metabolism^4,5,32,55,56^.

### Effects of AAV-GAA gene therapy on GAA^−/−^ mouse transcriptomics and Pompe disease signature

To understand the transcriptomic consequences of hGAA gene therapy in GAA KO mice, we applied liver depot gene therapy with an rAAV8 vector to induce liver-restricted expression and continuous secretion of hGAA into the blood and produce immune tolerance^57,58^, leading to increased muscle GAA activity and reduced glycogen content (Supplementary Fig. 6a, b). GSEA of AAV-treated and untreated KO mouse muscles showed that vector treatment caused significant (FDR < 0.15) improvement in oxidative phosphorylation (GO:0006119, *q* = 1.3 × 10^−2^; GO:0022900, *q* = 1.6 × 10^−2^), skeletal muscle contraction (GO:0003009, *q* = 1.5 × 10^−2^; GO:0036379, *q* = 1.5 × 10^−2^), and cholinergic muscle stimulation (GO:0007271, *q* < 1 × 10^−3^; GO:0042165, *q* = 2.4 × 10^−2^), and significantly (FDR < 0.15) reduced Golgi vesicle-mediated transport (GO:0006891, *q* = 5.3 × 10^−3^; GO:0006896, *q* = 2.4 × 10^−2^) and ubiquitin-related catabolism (GO:0071947, *q* = 6.5 × 10^−2^; GO:0031461, *q* = 2.5 × 10^−2^) (Supplementary Fig. 6d). These results suggested AAV therapy-induced reversal of multiple pathways affected by Pompe disease^5,32,53,59^. Specifically, of the 2244 genes that were significantly differentially expressed between KO and WT mice, 1006 (44.8%) were significantly reversed due to AAV treatment (Supplementary Fig. 6e). Transcriptome-wide analysis of the two comparisons (1: KO versus WT and 2: AAV-treated KO versus untreated KO) showed a negative correlation (Spearman’s correlation coefficient, −0.76; *p* < 2.2 × 10^−16^) (Supplementary Fig. 6f, g), further suggesting a reversal in the pathological transcriptional phenotype in GAA^−/−^ mice following AAV treatment. Changes in six of the affected genes were further validated using qPCR (Supplementary Fig. 6h). GSEA of AAV-treated versus untreated KO mice also showed significant reversal in the disease signature (Supplementary Fig. 6i, j) described above. Specifically, the gene set encompassing the genes upregulated in untreated KO mice were downregulated in AAV-treated KO mice (*q* < 1 × 10^−3^) and vice versa (*q* < 1 × 10^−3^) (Supplementary Fig. 6i, j).

### Transcriptomic comparison of healthy, IOPD, and rhGAA-treated IOPD myobundles

**Fig. 5:**
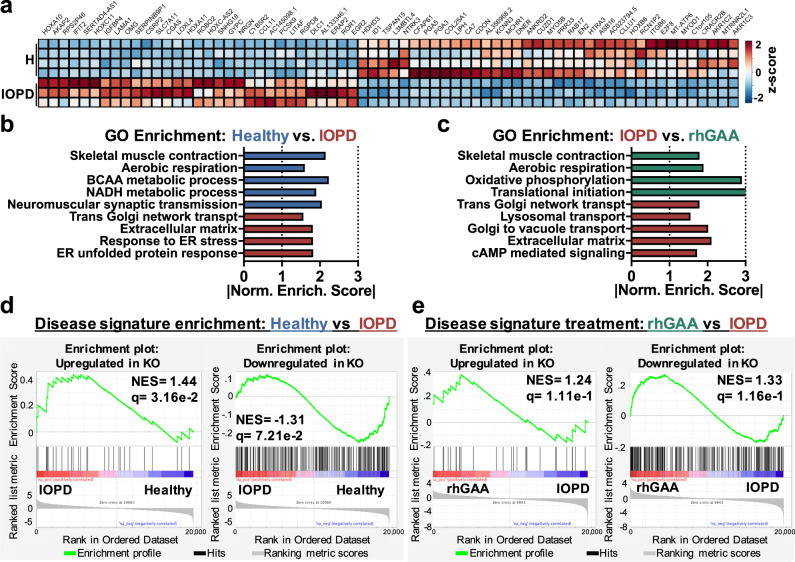
**Transcriptomic changes in myobundles as a result of IOPD and rhGAA treatment**. **a**, RNA-seq heatmap of genes with significantly different (p_adj_ < .05, |log_2_FC| ≥ 1) expression in IOPD vs. healthy myobundles. **b** GSEA on RNA-seq data to identify GO terms enriched in healthy (blue) or IOPD (red) myobundles plotted against normalized enrichment score (NES) (FDR < 0.15). **c** GSEA on RNA-seq data to identify GO terms enriched in rhGAA-treated (green) or untreated (red) IOPD myobundles (FDR < 0.15). **d** Enrichment plots of myobundles (IOPD vs. healthy) using disease signature gene sets consisting of the top 50% of the most significantly altered genes (*p*_adj_ < 0.05, |log_2_FC|≥2 in KO vs. WT RNA-seq), 39 of which were upregulated (left) and 243 downregulated (right) in KO vs. WT mice. **e** Enrichment plots of Pompe disease reversal in rhGAA-treated vs. untreated IOPD myobundles.

RNA sequencing analysis of myobundles was performed to further understand differences among healthy, IOPD, and rhGAA-treated IOPD myobundles and relate these findings to those obtained from GAA^−/−^ mice. When comparing healthy and IOPD myobundles, 68 genes exhibited significantly different gene expression (|log_2_FC| ≥ 1, *p*_adj_ < .05) (Fig. 5a). Five of these genes were selected for validation using qPCR, and showed the expected relationship between healthy and IOPD groups (Supplementary Fig. 7). Compared to healthy myobundles, IOPD myobundles exhibited increased expression of genes associated with extracellular matrix (GO:0031012, *q* = 4.5 × 10^−2^) and reduced expression of genes associated with skeletal muscle contraction (GO:0003009, *q* = 4.4 × 10^−3^), including those related to sarcomeric A-band (GO:0031672, *q* = 4.2 × × 10^−2^), I-band (GO:0031674, *q* = 3.8 × 10^−3^), and actin-mediated contraction (GO:0070252, *q* = 5.4 × 10^−3^) (Fig. 5b). In addition to association with contractile dysfunction, IOPD myobundles were associated with endoplasmic reticulum stress (GO:0034976, *q* = 4.3 × 10^−2^) and unfolded protein response (GO:1900101, *q* = 4.3 × 10^−2^), characteristic of misfolded protein accumulation (Fig. 5b)^60^. Interestingly, neuromuscular synaptic transmission (GO:0007274, *q* = 7.3 × 10^−3^) was also reduced in IOPD myobundles (Fig. 5b) and the constituent genes involved in the core enrichment of significant neuromuscular gene sets were confirmed to be highly represented in human skeletal muscle according to the Genotype-Tissue Expression (GTEx) database. This finding is consistent with the involvement of neuromuscular deficit in Pompe disease patients and mice^61,62^. Moreover, several metabolic pathways were also downregulated in IOPD myobundles, including aerobic (GO:0009060, *q* = 1.2 × 10^−1^), NADH (GO:0006734, *q* = 2.6 × 10^−2^), branched-chain amino acid (GO:0009081, *q* = 7.5 × 10^−4^), and pyruvate (GO:0006090, *q* = 4.9 × 10^−2^) metabolism. These findings are consistent with previous associations of IOPD with dysregulated mTOR signaling and reduced protein synthesis^4,5,63^, dysfunctional mitochondria and oxidative metabolism^39,53^, and muscle weakness. When collecting the GO terms identified in the healthy versus IOPD GSEA, examining individually significant genes (*p*_adj_ < 0.05) within these terms, and tracking their response to rhGAA treatment, significant reversal at the individual gene level was not observed (Supplementary Fig. 8). However, at the gene set level, comparing IOPD versus rhGAA-treated IOPD revealed that rhGAA treatment reversed the extracellular matrix term (GO:0031012, *q* = 6.5 × 10^−4^) while also being significantly associated with increased expression of genes involved in protein translation (GO:0006413, *q* < 1 × 10^−3^; GO:0006414, *q* < 1 × 10^−3^) and protein synthesis (GO:0002181, *q* < 1 × 10^−3^), endoplasmic reticulum function (GO:0072599, *q* < 1 × 10^−3^), oxidative metabolism (GO:0006119, *q* < 1 × 10^−3^) and aerobic respiration (GO:0009060, *q* = 6.8 × 10^−3^), and skeletal muscle contraction (GO:0003009, *q* = 1.7 × 10^−2^) (Fig. 5c).

### Disease signature enrichment in IOPD myobundles and attenuation with rhGAA therapy

To further determine whether myobundles recapitulated the disease phenotype resulting from complete loss of GAA in skeletal muscle, IOPD and healthy myobundles were tested for enrichment of the disease-specific transcriptional signature identified in mice (Supplementary Data 1). Interestingly, both up- and downregulated gene sets were significantly represented in IO Pompe myobundles (*q* = 3.16 × 10^−2^, *q* = 7.21 × 10^−2^) (Fig. 5d). Furthermore, we tested the ability of rhGAA treatment to reverse the mouse transcriptomic signature of disease in IOPD myobundles and found significantly reversed expression of the gene set downregulated in GAA^−/−^ mice (*q* = 1.16 × 10^−1^) but not upregulated in GAA^−/−^ mice (Fig. 5e). Consistent with the clinical studies^8,44^, these results suggest that rhGAA therapy yields only a partial rescue of IOPD phenotype in human skeletal muscle, signifying the need for improved treatment modalities.

## Discussion

In this study, we described the first in vitro three-dimensional human skeletal muscle model of infantile-onset Pompe disease, determined an IOPD disease signature in GAA^−/−^ vs. WT mice, and applied the developed IOPD myobundle model to study in vitro responses of human skeletal muscle to recombinant protein and AAV-based hGAA treatments. The IOPD myobundles exhibited low GAA activity and elevated glycogen content, consistent with previous studies in 2D cell culture^19,20,64^, GAA^−/−^ mice in vivo^45,65^, and clinical data^9,44^. Compared to 2D culture systems, 3D tissue-engineered skeletal muscle permits extended culture time, formation of more mature myofibers, and assessment of muscle function^21^. The ability to measure contractile force, in particular, allowed us to identify metabolic stress conditions that revealed distinct functional responses (tetanic force production, fatigue resistance) between healthy and IOPD myobundles. The reproducibility and robustness of these results were ensured using muscle cells from multiple patients and healthy donors. We further validated this human in vitro model by performing transcriptomic analysis which showed that IOPD myobundles exhibited reduced expression of genes involved in skeletal muscle contraction and increased expression of genes involved in endoplasmic reticulum stress, consistent with the transcriptomic changes found in GAA^−/−^ vs. wild-type mice. In vitro rhGAA therapy in myobundles partially reversed the transcriptomic disease signature identified in mice without correcting stress-revealed functional deficit. Together, these results establish a new in vitro human skeletal muscle model to improve understanding and treatment of Pompe disease.

In IOPD, GAA deficiency results in glycogen buildup in muscle fibers, followed by lysosome enlargement and rupture^44^. In our system, after 2 weeks of 3D differentiation, GAA-deficient IOPD myobundles showed 3-fold greater glycogen content relative to healthy myobundles (reaching levels measured in Pompe muscle biopsies^66^), along with significantly increased lysosomal area and Lamp2 expression, indicative of lysosome enlargement (Fig. 2d–g). A secondary consequence of lysosome dysfunction is impaired autophagy, which leads to autophagosome buildup, accumulation of ubiquitinated proteins^6^, and disruption of muscle architecture^67^. Based on assessment of the LC3-II/LC3-I ratio, autophagosome accumulation was not observed in IOPD myobundles, consistent with findings in cultured IOPD skeletal muscle cells^64^ and cardiomyocytes^59^. While the predominance of autophagosome buildup was found in isolated muscle fibers from LO rather than IOPD patients^68^, a separate study showed increased LC3-II and p62 expression in IOPD vs. healthy muscle^69^, suggestive of impaired autophagy and protein aggregation. Our GSEA analysis revealed that IOPD myobundles exhibited pathological upregulation of gene sets involved in endoplasmic reticulum stress, previously shown to induce autophagy in LOPD patient fibroblasts^60^. Moreover, 24 h treatment with chloroquine (which blocks autophagosome-lysosome fusion and further disrupts lysosome function^37^) revealed a greater vulnerability to functional loss in IOPD than healthy myobundles (Fig. 3a–c). Furthermore, given that IOPD patients require a median time of 4 months to develop muscle weakness^70^, these results suggest that IOPD myobundles may require a longer culture time and application of various metabolic challenges^4,71,72^ to accelerate the acquisition of an advanced pathological phenotype with significant autophagic buildup.

Given that extensive autophagosomes can directly impede myofiber contractions^73,74^, the lack of autophagic buildup in IOPD myobundles likely explains the lack of contractile deficit observed under baseline culture conditions (Fig. 1n), which could be further attributed to the immaturity of myobundles relative to native muscle, evident from their expression levels of myosin heavy chain isoforms and calcium-handling genes^24^. Nevertheless, under baseline conditions IOPD myobundles exhibited downregulation of gene sets involved in skeletal muscle contraction and sarcoplasmic reticulum function^1,39^ (Fig. 5b), similar to a microarray study of muscle biopsies from IO Pompe and healthy donors^75^. Interestingly, examining the core enrichment for the muscle contraction gene set (GO:0006941) in healthy versus IOPD myobundles revealed differentially expressed genes related to muscle excitability (*SCN5A, SCN3B, KCNE2*), neuronal excitation (*CHRNA1, CHRNB1, CHRND*), and Na^+^/K^+^ ATPase (*ATP1A1, ATP1A2*). These findings suggest that replicating at least some aspects of advanced Pompe disease in vitro may require functional innervation^76^, as achieved in co-cultures of engineered muscle and motoneurons^77^.

Current enzyme replacement therapy (ERT) for Pompe disease consists of intravenous delivery of rhGAA (Myozyme®/Lumizyme®; alglucosidase alfa), which increases muscle GAA activity, reduces glycogen accumulation, and improves muscle function and patient survival^9,44,78^. Consistent with clinical results and previous in vitro studies^64,79^, 7-day rhGAA treatment yielded successful enzyme uptake (Fig. 4b, c) and strong reduction of glycogen content in IO Pompe myobundles (Fig. 4e), but unlike long-term clinical ERT^80^, it only modestly increased GAA activity (Fig. 4d), primarily via improved abundance of the mature 70-kDa GAA isoform (Fig. 4b, c). Interestingly, rhGAA treatment of healthy myobundles did not increase expression of any GAA isoforms (Supplementary Fig. 4a, b), which indicates the potential existence of a negative feedback regulating GAA protein level. The incomplete therapeutic effects induced by modestly increased GAA activity were also evident from the lack in reversal of lysosomal accumulation or increased vulnerability to GP inhibition and chloroquine (Fig. 4k, l). While rhGAA treatment was unable to significantly correct the expression of individual genes identified within the enriched GO terms of IOPD versus healthy myobundles (Supplementary Fig. 8), gene set analysis showed rhGAA treatment significantly upregulated gene sets known to be downregulated in disease (related to oxidative metabolism^39,53^, muscle contraction, and protein synthesis^4^ pathways) (Fig. 5c). Studies in murine GAA KO myotubes in vitro and GAA^−/−^ mice in vivo suggested that lysosome size reduction occurs in a dose-dependent manner and requires high rhGAA concentrations^81^, while several weeks of ERT may be needed to fully rescue dysregulated glycogen metabolism^82^. Accordingly, re-normalization of GAA activity, lysosome abundance, and sensitivity to GP inhibition and chloroquine in IOPD myobundles may require increased duration and dose of rhGAA treatment or development of novel means to improve rhGAA affinity for^83^ or expression of^45^ mannose-6-phosphate receptor, which mediates muscle uptake of extracellular GAA.

Interestingly, rAAV9-MHCK7hGAA transduction of IOPD myobundles produced levels of GAA activity comparable to those of healthy myobundles (Supplementary Fig. 5b), but despite glycogen clearance (Supplementary Fig. 5c), it did not improve contractile response to GP inhibition or chloroquine (Supplementary Fig. 5e, f). While complete functional recovery of muscle may be achieved with longer AAV vector treatment, it may also require the effective rescue of GAA activity in non-muscle cells which in myobundles were not transduced using the MHCK7 promoter. For example, fibroblasts in Pompe disease are also affected by GAA deficiency, showing glycogen accumulation, increased sensitivity to endoplasmic reticulum stress-induced autophagy, and impaired extracellular uptake of rhGAA^60,84^ and may contribute to muscle pathology via paracrine or juxtacrine effects. Alternatively, although unlikely, the three IOPD donors used in this study may not represent the typical functional response to rhGAA or rAAV9 vector therapy, given the range of clinical outcomes observed in response to ERT^8,44^.

Our RNA-Seq analyses of GAA^−/−^ and WT mouse quadriceps provided further mechanistic insights in Pompe disease, while allowing us to define a disease-specific transcriptomic signature for additional validation of the human model. Consistent with previous studies^39,53^, GAA^−/−^ quadriceps showed significant upregulation of L-type calcium channel subunits (e.g., *Cacng7* (Supplementary Fig. 6h), *Cacna1c*, and *Cacna2d4*) and downregulation of gene sets involved in oxidative metabolism and mitochondrial function (Supplementary Fig. 6c). Furthermore, GAA^−/−^ muscle exhibited downregulation of protein synthesis gene sets (Supplementary Fig. 6c), consistent with the muscle atrophy and protein loss observed in GAA^−/−^ mice and Pompe disease patients where post-translational modifications may serve to further regulate cellular protein metabolism^85^. Changes in other less-investigated pathways suggested additional mechanisms underlying Pompe disease, including negative regulation of Notch (involved in GAA expression^86^), increased cAMP (involved in glycogen metabolism^87^), and increased PKC signaling. A Pompe disease signature composed of the top significantly altered genes (upregulated and downregulated; Supplementary Data 1) was largely reversed by liver-specific AAV-hGAA treatment (Supplementary Fig. 6e–j), which increased enrichment of oxidative phosphorylation, synaptic transmission, and muscle contraction gene sets (Supplementary Fig. 6d). Importantly, human IOPD myobundles also exhibited significant enrichment for altered gene sets present in the mouse disease signature (Fig. 4d) and consistent with histological and functional results, showed a partial reversal of the signature gene sets after 7-day rhGAA treatment (Fig. 4e). Along with studies in clenbuterol-treated LOPD patients^88^, our results confirmed the utility of RNA-Seq analysis to understand treatment effects in Pompe disease.

In summary, we introduce an in vitro 3D human skeletal muscle model of infantile-onset Pompe disease that displays: (1) morphological and biochemical features observed in patients’ biopsies, (2) contractile deficits revealed under metabolic stress, (3) transcriptomic signature consistent with that of a mouse model, and (4) partial phenotype rescue with in vitro recombinant protein or AAV-mediated treatments. We anticipate that with further development, human IOPD myobundles will hold the potential to serve as a novel pre-clinical platform for mechanistic studies and therapeutic testing in Pompe disease.

## Methods

### Human myoblast isolation and expansion

Ten human skeletal muscle samples were obtained from standard needle biopsies or surgical discards using protocols approved by the Duke University Institutional Review Board. Of the healthy muscle donors 1–5 (12/F, 16/M, 15/M, F/18, F/14 (age/sex), respectively), the largest biopsies and cell yields were obtained for donors 1–3 and were consequently used in all performed assays. Muscle samples from healthy donors 4 and 5 were only used in select assays (GAA activity, glycogen content, RNA sequencing). The IOPD samples (A, B, C) came respectively from 1 male (6 months old, CRIM-negative), and 2 female (7 years old, CRIM-negative; 13 years old, CRIM-positive) patients. The LOPD samples (a, b) came from 1 male (46 years old; c.-32-13T>G, c.2238G>A) and 1 female (30 years old; c.-32-13T>G, c.2481+102_2646+31) patient. Human skeletal muscle cells were isolated as described previously^22,23^. Briefly, muscle samples were washed in PBS, minced, and enzymatically digested in 0.05% trypsin for 30 min. After FBS neutralization of the digestion buffer, muscle pieces were centrifuged, resuspended in growth media (GM; low-glucose DMEM, 10% FBS, dexamethasone, EGF, and 1× antibiotic-antimycotic), and pre-plated for 2 h to reduce fibroblast contamination. Unattached muscle fragments were then gathered and replated on Matrigel (BD Biosciences)-coated flasks for outgrowth and cell expansion (passage 0) in GM. Cells were frozen at p2, p3, and p4 in 90% FBS and 10% DMSO and stored in liquid nitrogen. After the fifth passage, cells were incorporated into a fibrin-based gel for myobundle construction.

### Fabrication and culture of tissue-engineered human myobundles

Myobundles were formed within polydimethylsiloxane (PDMS) molds containing two semi-cylindrical wells (7-mm long, 2-mm diameter), cast from 3D-machined Teflon masters^23^. PDMS molds were coated with 0.2% (w/v) pluronic F-127 (Invitrogen) for 1 h at room temperature to prevent hydrogel adhesion. Laser-cut Cerex frames positioned around the two wells were used to anchor the ends of the myobundles and facilitate handling. A cell solution (7.5 × 10^5^ cells in 17.2 μL media per bundle + 2 μL of 50 U/mL thrombin in 0.1% BSA in PBS) and a gelling solution (3 μL media + 10 μL Matrigel + 10 μL of 15 mg/mL fibrinogen in PBS) were prepared in separate vials on ice for up to eight myobundles per vial. Gelling solution was added to the cell solution, mixed thoroughly, and injected into the PDMS wells to polymerize at 37 °C for 30 min. Resulting myobundles were cultured dynamically on a rocker (0.5 Hz) in GM supplemented with 1.5 mg/mL 6-aminocaproic acid (ACA, Sigma) for 4 days. The media was then switched to differentiation media (DM) consisting of a custom low-glucose, low-amino acid media^89^ to mimic physiological amino acid concentrations, with 1× N2 serum supplement (Thermo Fisher), and 2 mg/mL ACA. The media was exchanged every other day.

### Metabolic stress induction in myobundles

Both healthy and IOPD myobundles were exposed to 24 h small molecule treatment between differentiation days 13 and 14. Glycogen phosphorylase was inhibited using 10 µM CP-91,149 (Cayman) and lysosomal stress was induced using 5 µM chloroquine (Sigma, C6628). For 24 h glucose starvation, myobundles were cultured in low-amino acid media with N-2 supplement (as described above) without glucose. All biochemical and functional measurements were performed at the end of the 24 h stress period.

### Recombinant GAA treatment of myobundles

Recombinant human acid alpha glucosidase (rhGAA, alglucosidase alfa, Myozyme®/Lumizyme®) was obtained from Sanofi Genzyme and added at 2 µg/mL between differentiation weeks 1 and 2 during every-other-day media change. Untreated control received the application of media only.

### AAV vector preparation and treatment of myobundles

AAV vectors were prepared as described previously and purified by centrifugation with a cesium chloride gradient^65^. Briefly, HEK293 cells were transfected with an AAV vector plasmid, the packaging plasmid^90^, and pAdHelper (Stratagene, La Jolla, CA). After 48 h, cells were harvested and freeze-thawed three times. AAV vectors were isolated by sucrose cushion pelleting, followed by two cesium chloride gradient centrifugation steps. AAV stocks were dialyzed against three changes of phosphate-buffered saline with 5% sorbitol added to the third dialysis, and aliquots were stored at −80 °C until use. The viral vectors were handled under the guideline of Biohazard Safety Level 2 which is published by the NIH. For myobundle experiments, IOPD myoblasts were mixed with either rAAV9-MHCK7eGFP (control) or rAAV9-MHCK7hGAA at 2.5 × 10^3^ vector genomes/cell during myobundle fabrication.

### Isometric force measurements in myobundles

Contractile properties of myobundles were measured using a custom, temperature-controlled force measurement setup described previously^22,23^. Briefly, single myobundles were anchored on one end to an immobilized PDMS block and on the opposite end to a suspended PDMS float connected to a force transducer. Tissues were stimulated using 40 V/cm, 5 ms electrical pulses via parallel platinum electrodes to generate twitch (single pulse) and tetanic (20 Hz for 1 s) contractions. A computer-controlled motorized linear actuator (Thor labs) connected to the PDMS float was used to stretch myobundles to a length (1.12 × culture length) that yielded the maximum generated force. Recorded force traces were analyzed for peak twitch or tetanus amplitude, time to peak twitch, and half-relaxation time using a custom MATLAB program^22,23^.

### GAA activity assay

Individual myobundles were resuspended in 180 µL of water plus protease inhibitor (Sigma) and were lysed using the Bullet Blender (Next Advance). Protein concentration was measured using a BCA protein assay. To measure GAA activity, 15 µL of sample, 2.14 µL of 70 mM 4-MU-a-D (Sigma M9766), and 82.86 µL of citrate-phosphate buffer (pH = 3.7) were incubated in a 96-well plate at 37 °C for 1 h. Stop buffer (200 µL of 0.5 M EDTA buffer pH = 11.8) was then added to halt the reaction, and fluorescence at 450 nm was measured using a plate reader. 4MU (Sigma M1381)-containing wells with concentrations from 100 nM to 20 µM 4MU were run in parallel to generate a standard curve for calculating product generation during the reaction. 100 µL of each standard was added prior to incubation, and 200 µL stop buffer was added to each well after incubation, diluting the concentration of the standard 1:3. As a baseline for zero activity, a 15 µL water sample was incubated with 2.14 µL of 70 mM 4MU-a-D and 82.86 µL of citrate-phosphate buffer.

### Glycogen content assay

Samples were isolated as described for the GAA activity assay, diluted 1:10 in sodium acetate buffer (0.1 M, pH 5.5), and heated at 100 °C for 3 min to inactivate endogenous amylases. Each sample was distributed into two wells with 50 µL sample in each. One µL of 120 U/mL amyloglucosidase (Sigma Aldrich, cat. 10113) was added to one well for 45 min. Both wells then received a solution containing 1 µL of 100 U/mL glucose oxidase (Sigma Aldrich, cat. G6125), 1 µL of 10 U/mL horseradish peroxidase (Sigma Aldrich cat. 516531), 0.5 µL of 10 mM ADHP (Setareh Biotech, cat. 6773), and 47.5 µL of PBS for 30 min. Glycogen standards (Cayman Chemical, cat. 700481) were generated and treated in parallel to produce standard curves for each assay. Absorbance at 560 nm or fluorescence at 540/590 nm for low-concentration samples was measured as a readout.

### Immunohistochemistry

Cell monolayers were fixed in 4% paraformaldehyde in PBS for 10 min and myobundles were fixed in 2% paraformaldehyde in PBS overnight at 4 °C^91^. Following fixation, samples were washed in PBS followed by blocking in 5% chick serum with 0.3% Triton-X 100. Primary and secondary antibodies were applied overnight at 4 °C. Images were acquired using the Zeiss 880 inverted confocal microscope. For live imaging of lysosomes, lysotracker was incubated at 1:10,000 lysotracker (Thermo Fisher) for 15 min and washed in PBS before imaging. For cross-sectional images, fixed tissues were cut in half, frozen in OCT blocks using liquid nitrogen, and cut at 10 µm increments. Primary and secondary antibody information can be found in Supplementary Table 1.

### Western blotting

Western blotting was performed as previously described^22,23^. Briefly, protein was isolated in RIPA lysis and extraction buffer with protease inhibitor (Sigma) and phosphatase inhibitor cocktail (Roche), lysed using a Bullet Blender (Next Advance), and protein concentration was determined using a BCA assay (Thermo Fisher). Western blots were performed using Bio-Rad Mini-PROTEAN gels and transferred using a Pierce 2000 powerblot semi-dry transfer system (Thermo Fisher). Primary antibodies were applied overnight at 4 °C before application of HRP-conjugated anti-mouse (1:20,000) and HRP-conjugated anti-rabbit antibody (1:5000; SCBT). Chemiluminescence was performed using Clarity Western ECL substrate (Bio-Rad). Images were acquired using a Bio-Rad Chemidoc and analyzed using Image Studio. Original blots are included in Supplementary Fig. 9. Samples were first normalized to GAPDH expression. In healthy versus IOPD comparisons, samples were normalized a second time such that the mean of healthy means was 1. In rhGAA-treated versus untreated IOPD comparisons, the rhGAA group was normalized to its respective IOPD donor control.

### Flow cytometry

Muscle cells expanded for five passages were fixed in 4% paraformaldehyde for 10 min and stained with CD56 PE-Cy7 (1:100, BD Biosciences) and CD29 APC (1:100, BD Biosciences) in 3% FBS for 30 min on ice followed by three PBS wash steps. Samples were run on a BD FACSCanto II and analyzed in FlowJo. Cells were gated based on size and granularity to exclude debris, followed doublet removal, before recording CD56 and CD29 intensities. Additional antibody information can be found in Supplementary Table 1.

### GAA-KO mice

All animal procedures were performed under the guidelines of the Duke University Institutional Animal Care and Use Committee. Two-month-old GAA-KO male mice with a C57BL/6 background were administrated the liver-specific hGAA expression vector AAV2/8-LSPhGAApA^57^ (2 × 10^12^ vg/kg) via tail vein injection. Control treatment group consisted of mice injected with PBS. After 4 months, the mice were euthanized and the quadriceps were harvested, along with the quadriceps of age-matched wild-type C57BL/6 mice. The collected quadriceps were split for RNA extraction and for assaying GAA activity and glycogen content^58,65^.

### RNA extraction and quantitative RT-PCR

RNA was isolated from mouse tissue and human myobundles using the Bullet Blender (Next Advance) and the RNeasy Fibrous Tissue Mini Kit (Qiagen), following by RNA reverse-transcription using the iScript cDNA Synthesis Kit (Bio-Rad). Quantitative RT-PCR for muscle-related genes was performed with iTaq Universal SYBR Green Supermix (Bio-Rad) according to the manufacturer’s instructions. Primer information can be found in Supplementary Table 2.

### RNA sequencing and gene set enrichment analysis (GSEA)

Two RNA-seq datasets (mouse and human) were generated from isolated mRNA. The samples were processed by the Duke Center for Genomic and Computational Biology in the same manner unless otherwise specified. RNA-seq data were processed using the fastp toolkit^92^ (human) or TrimGalore toolkit^93^ (mouse) to trim low-quality bases and Illumina sequencing adapters from the 3′ end of the reads. Only reads that were 20 nt or longer after trimming were kept for further analysis. Reads were mapped to the human (version GRCh38v93) or mouse (version GRCm38v73) genome and transcriptome^94^ using the STAR RNA-seq alignment tool^95^. Reads were kept for subsequent analysis if they mapped to a single genomic location. Gene counts were compiled using the featureCounts tool^96^ (human) or HTSeq tool^97^ (mouse). Only genes that had at least 10 reads in any given library were used in subsequent analysis. Normalization and differential expression were carried out using the DESeq2^98^ Bioconductor^99^ package within the R statistical programming environment. For the human Pompe myobundle rhGAA-treated versus untreated analysis, we included personID as a cofactor in the model. The false discovery rate was calculated using the Benjamini-Hochberg procedure to control the false-positive rate during multiple hypothesis testing. Gene set enrichment analysis^100^ was performed to identify gene ontology terms and pathways associated with altered gene expression for each of the comparisons performed with FDR cutoff 0.15. The Pompe disease signature was generated using the top 50% of differentially expressed genes with |log_2_FC| ≥ 2 and *p*_adj_ < 0.05 in the mouse WT versus GAA^−/−^ comparison, and these genes were then divided based on up- or downregulation (Supplementary Data 1a, b). To test the enrichment of this signature in the human myobundle system, mouse genes were first mapped to human orthologs using the biomaRt^101^ package in R, which was followed by GSEA^100^. The human orthologs (HGNC symbols) are listed alongside the mouse disease signature in Supplementary Data 1a, b.

### Statistics and reproducibility

Data are expressed as mean ± SEM. Unless noted otherwise, statistical comparisons were made using the mixed-effects model *Y*_*ij*_ = *α*_*i*_ + *β* + *ε*_*ij*_, where *Y* is the response variable for the *j*th measurement of individual *i*, *α*_*i*_ is the random effect for individual *i*, *β* is the fixed effect (i.e., IOPD vs. healthy or treated vs. untreated IOPD), and random error (*ε*_*ij*_). Significance for the fixed effects was calculated using a Wald test for *p* < 0.05 using the lme4 package in R.

### Reporting summary

Further information on research design is available in the Nature Research Reporting Summary linked to this article.

## Supplementary information

Supplementary Information

Description of Additional Supplementary Files

Supplementary Data 1

Reporting Summary

## Data Availability

Data supporting the results of the main figures are available at 10.6084/m9.figshare.14173013.v1^102^.The sequencing data generated for this study have been deposited in Gene Expression Omnibus under the SuperSeries GSE159064 and SubSeries GSE159062 (human myobundle model) and GSE159063 (GAA KO mouse model). Data referenced in the “Discussion” of this study are available on Gene Expression Omnibus GSE57980 and GSE38680. Any remaining information can be obtained from the corresponding author upon reasonable request.
