# Supplementary material for: Three-dimensional tissue-engineered human skeletal muscle model of Pompe disease
Source: Commun Biol. 2021 May 5;4:524. doi: 10.1038/s42003-021-02059-4 (PMC8100136; doi:10.1038/s42003-021-02059-4)
Supplement: Supplementary file 1 — Supplementary Information [file 42003_2021_2059_MOESM1_ESM.pdf]

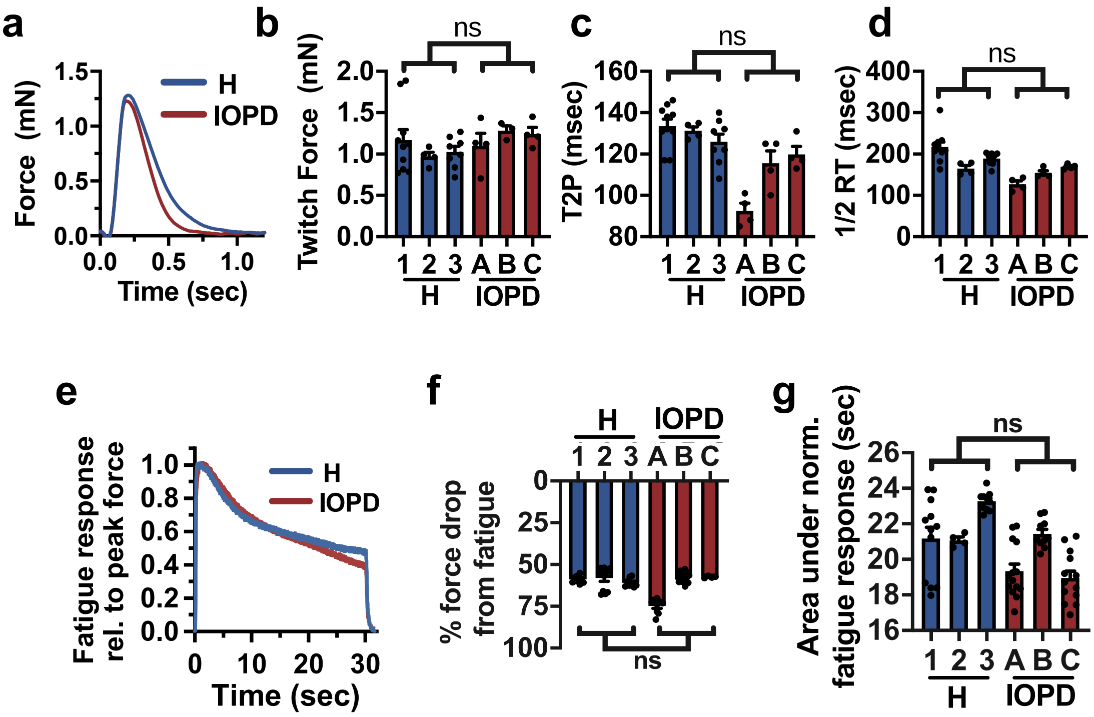

Supplementary Figure 1. Twitch and fatigue force response in healthy and IOPD myobundles. a-d, Representative twitch force trace from healthy (blue) and IOPD (red) myobundles (**a**) and quantified twitch force (**b**), time to peak tension (T2P, **c**), and half-relaxation time (1/2 RT, **d**) (n=4–10 myobundles per donor) from 3 healthy (1, 2, 3) and 3 IOPD (A, B, C) donors. **e,** Representative force traces during fatigue test (20 Hz stimulation for 30 sec) normalized to value of peak force. **f,g,** Quantified (**f**) percent force decline at the end of fatigue stimulation (n=4–20 myobundles per donor), and (**g**) area under normalized force curve during fatigue stimulation (n=4–14 myobundles per donor). Data: mean ± SEM. ns, not significant.

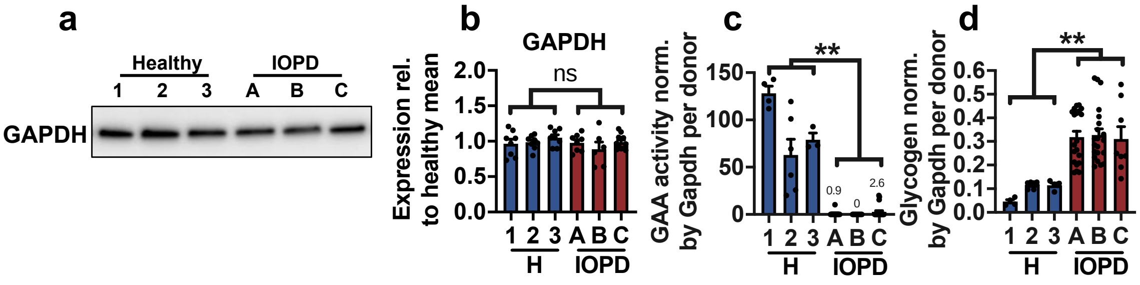

Supplementary Figure 2. GAA activity and glycogen content in healthy and IOPD myobundles normalized by GAPDH. a-b, Representative Western blots (**a**) and quantified protein expression (**b**) of GAPDH (n=6-12 myobundles per donor) from 3 healthy (1, 2, 3) and 3 IOPD (A, B, C) donors. **c-d,** GAA activity (**c**) and glycogen content (**d**) in 3 healthy (1, 2, 3) and 3 IOPD (A, B, C) donors (n=4–21 myobundles per donor) normalized by mean GAPDH expression per donor. Data: mean ± SEM. $^{**}p < 0.01$; ns, not significant.

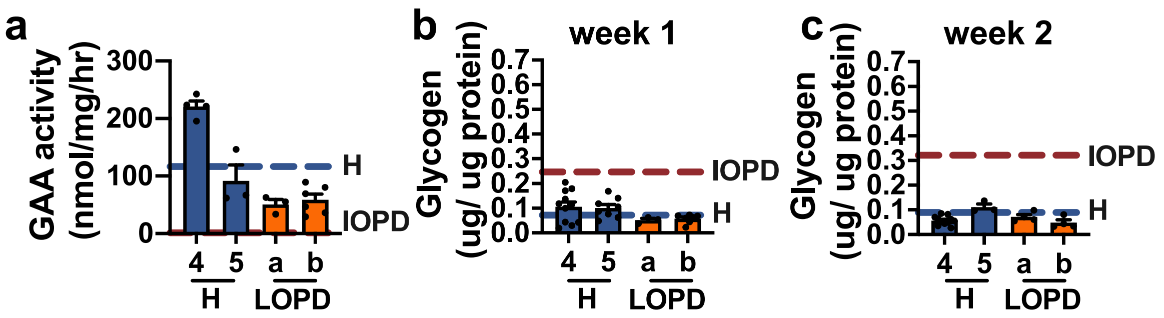

Supplementary Figure 3. GAA activity and glycogen content in late-onset Pompe disease (LOPD) myobundles. a, GAA activity in 2-week differentiated myobundles engineered from two additional healthy donors, 4 and 5, and two donors with LOPD, a and b (n=3–6 myobundles per donor). **b-c,** Glycogen content in myobundles from healthy donors 4 and 5 and from LOPD donors a and b after one week (**b**) and two weeks (**c**) of 3D differentiation (n=3–11 myobundles per donor). Dashed H-line denotes the mean value across all five healthy donors, 1-5, and dashed IOPD-line denotes the mean value across all three untreated IOPD donors, A-C.

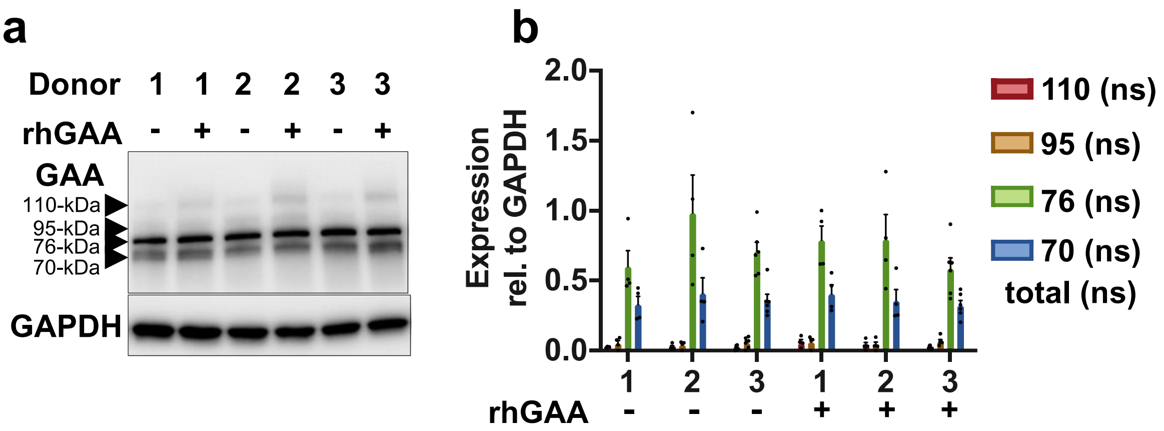

Supplementary Figure 4. rhGAA treatment of healthy myobundles. a, Representative Western blot of GAA isoforms in rhGAA treated (+) and untreated (-) myobundles from 3 healthy donors (1, 2, 3) and quantification of isoform expression relative to GAPDH (**b**, n=4-6 myobundles per donor). Data: mean ± SEM. ns, not significant between + and – group across the donors.

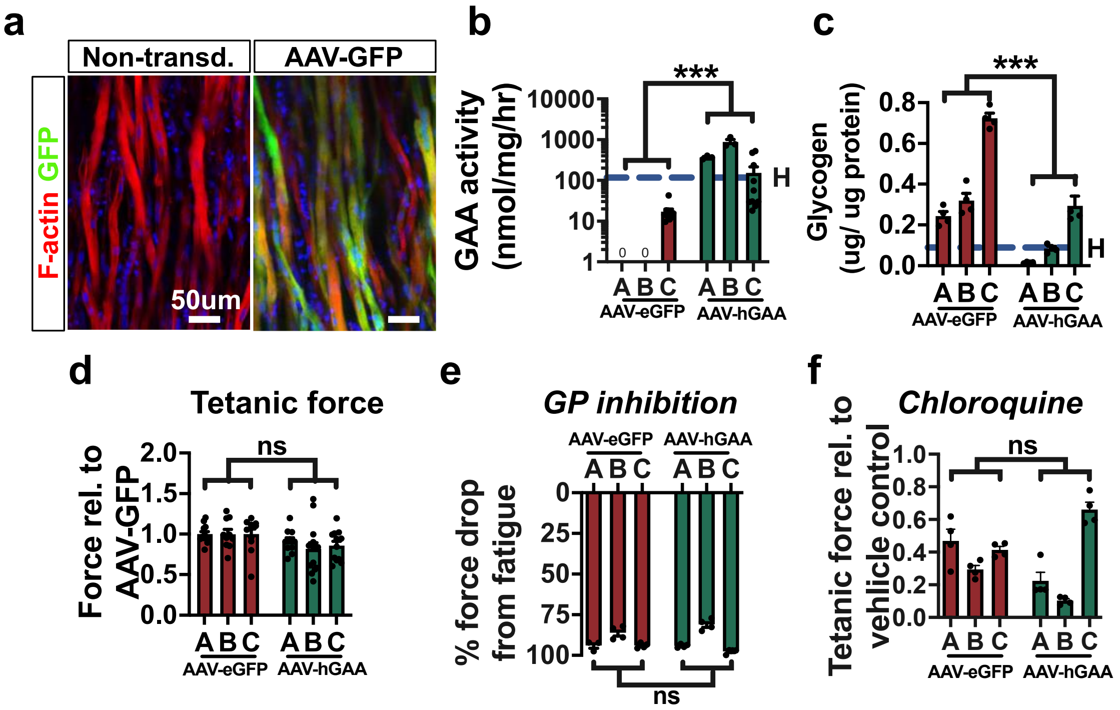

Supplementary Figure 5. hGAA gene therapy in IOPD myobundles. a, Representative images of rAAV9-MHCK7eGFP (AAV-GFP) vector-transduced and non-transduced IOPD myobundles differentiated for 2 weeks and stained with GFP antibody. **b-c,** Quantified GAA activity (**b**, n=3–10 myobundles per group) and glycogen content (**c**, n=4 myobundles per group) in 2-week-differentiated IOPD myobundles transduced with AAV-GFP or rAAV9-MHCK7hGAA (AAV-hGAA) vectors from 3 IOPD donors (A, B, C). Dashed H-line denotes mean value across all five healthy donors. **d,** Tetanic force of IO myobundles after AAV-hGAA vector treatment shown relative to AAV-GFP vector treatment (n=10–14 myobundles per group). **e,** Percent force decline at the end of fatigue stimulation in response to 24 h of GPi exposure in AAV-GFP or AAV-hGAA vector treated IOPD myobundles (n=3–4 myobundles per group). **f,** Tetanic force in response to 24 h of chloroquine exposure in AAV-GFP or AAV-hGAA vector treated IOPD myobundles (n=4 myobundles per group). Data: mean ± SEM. ***p < 0.001; ns, not significant.

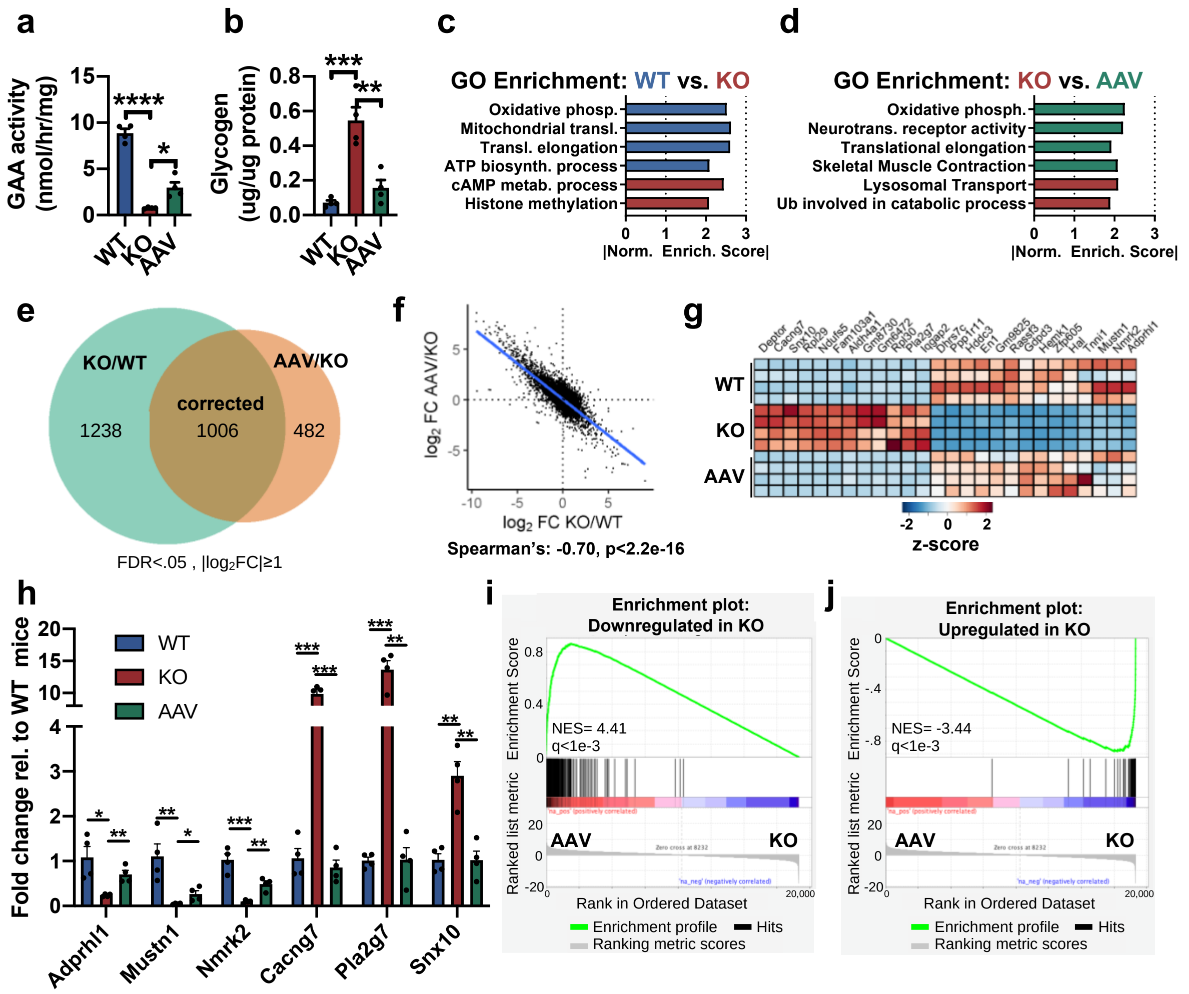

Supp. Fig. 6. Transcriptomic changes in mice as a result of GAA knockout and hGAA gene therapy. a,b, GAA activity (**a**) and glycogen content (**b**) measured in quadriceps of wild-type (WT) mice, GAA$^{-/-}$ (KO) mice, and liver-specific AAV2/8-LSPhGAApA (AAV) vector-treated KO mice (n=4 mice per group). **c,** GSEA on RNA-seq data to identify GO terms enriched in WT (blue) or KO (red) mice plotted against normalized enrichment score (NES) (FDR<0.15). **d,** GSEA on RNA-seq data to identify GO terms enriched in KO (red) or AAV vector-treated (green) mice (FDR<0.15). **e,** Venn diagram with differentially expressed genes (p_{adj}<.05, |$\log_2$FC|≥1) in two comparisons (1: KO vs. WT mice; 2: AAV vector-treated vs. untreated KO mice), with the intersection indicating significantly reversed genes following AAV vector treatment. **f,** Spearman's correlation of the AAV/KO vs. KO/WT whole transcriptome. **g,** Heatmap of 26 most significantly reversed genes following AAV vector treatment. **h,** qPCR validation of six reversed genes normalized to housekeeping gene *B2m* and shown as fold-change relative to WT. Welch's 2-sided t-test was performed to compare WT vs. KO mice, and AAV vector-treated vs. untreated KO mice. Data: mean ± SEM. *$p < 0.05$, **$p < 0.01$, ***$p < 0.001$ (n=4 mice per group). **i,j,** Enrichment plots on AAV vector-treated vs. untreated KO mice using disease signature gene sets consisting of the top 50% most significantly altered genes (p_{adj}<.05, |$\log_2$FC|≥2 in KO vs. WT RNA-seq), 243 of which were downregulated (**i**) and 39 upregulated (**j**) in KO vs. WT mice.

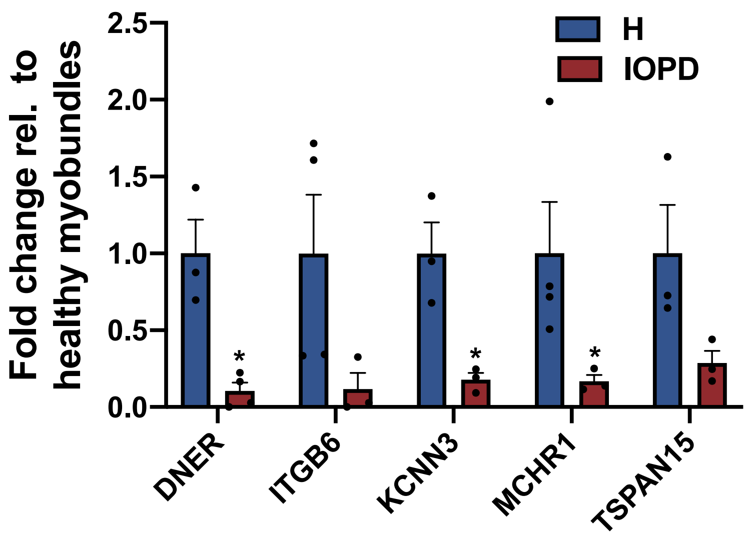

Supplementary Figure 7. qPCR validation of RNA-seq results in myobundles. Five genes from the RNA-seq dataset with significant difference in expression between healthy and IOPD myobundles validated using qPCR. The expression is normalized to housekeeping gene *B2M* and shown as fold-change relative to healthy myobundles (n=3–4 donors per group). Data: mean ± SEM. $^*p < 0.05$.

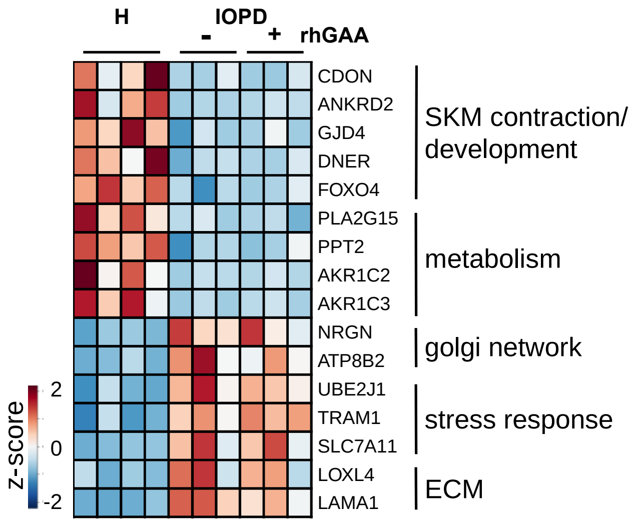

Supplementary Figure 8. RNA-seq heatmap for healthy, IOPD, and rhGAA-treated myobundles. Heatmap of genes expressed in healthy, IOPD, and rhGAA-treated IOPD myobundles, identified in healthy versus IOPD GO term enrichment analysis of RNA-seq data with $p_{adj}<.05$. (n=3-4 donors per group).

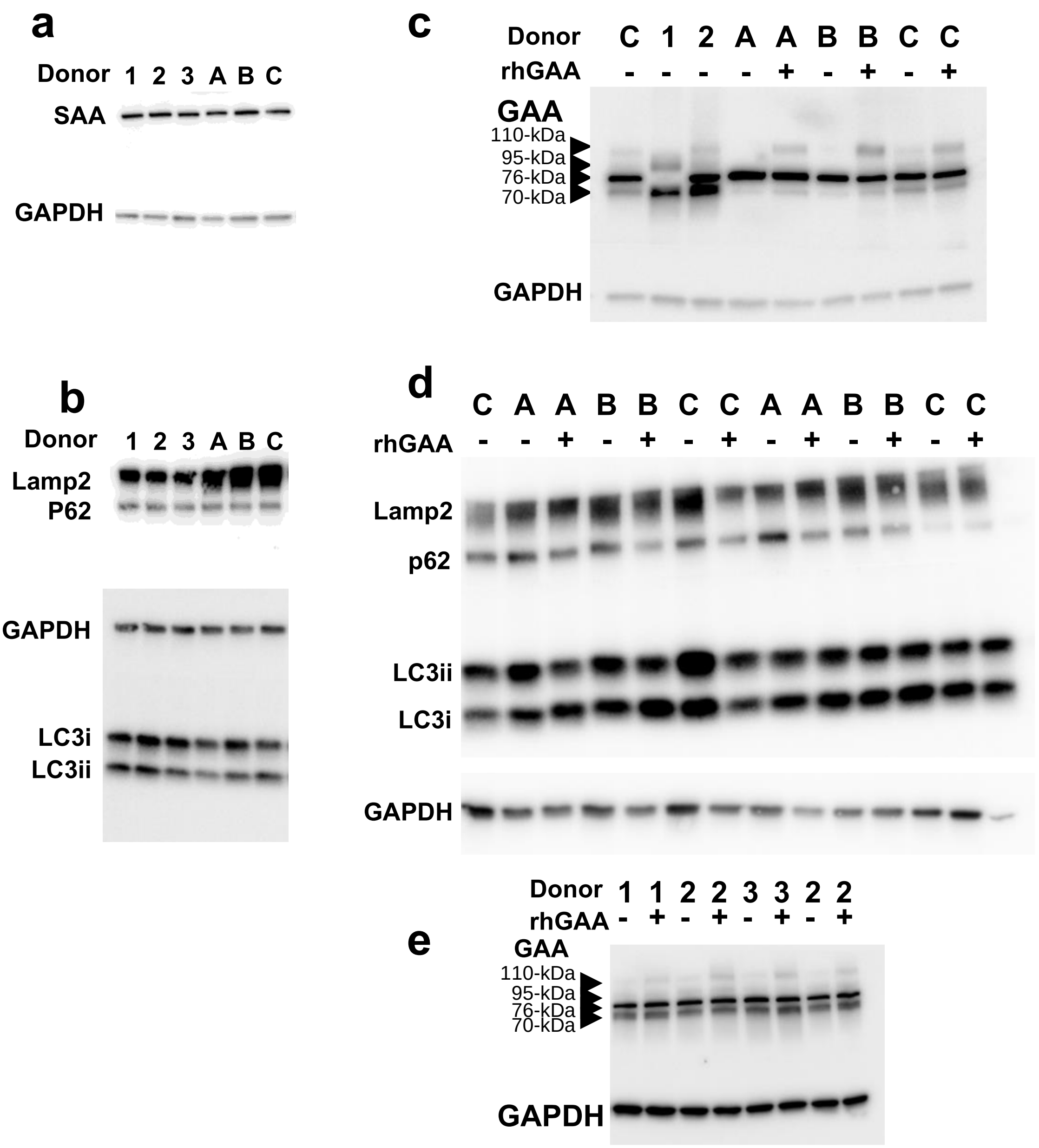

Supplementary Figure 9. Original Western blots. Western blots for healthy (1, 2, 3) and IOPD (A, B, C) myobundles with and without rhGAA treatment (+, -) **a,** Western blot for Figure 1l. **b,** Western blot for Figure 2f for first stain (Lamp2, p62) and second stain (Gapdh, LC3i, LC3ii)**. c,** Western blot for Figure 4b. **d,** Western blot for Figure 4f for first stain (Lamp2, p62, LC3ii, LC3i) and second stain (Gapdh). **e,** Western blot for Supp. Fig. 4.

Antibody	Company	Product No.	Dilution
SAA	Sigma	A7811	IF: 1:200, WB: 1:1000
GAPDH	SCBT	SC-47724	WB: 1:1000
DAPI	ThermoFisher	62247	IF: 1:300
Pax7	DSHB	PAX7-b	IF: 1:100
Myogenin	SCBT	sc-576	IF: 1:100
Myomesin	DSHB	mMaC-myomesin-B4	IF: 1:200
Lamp2	SCBT	sc-18822	WB: 1:1000
LC3	Sigma	L7543	WB: 1:2000
p62/SQSTM1	Cell Signaling Tech	5536S	WB: 1:1000 (BSA)
GAA	Abcam	ab137068	WB: 1:1000
Lysotracker Red	ThermoFisher	L7528	IF: 1:20000
Phalloidin 488	ThermoFisher	A12379	IF: 1:300
GFP	Abcam	ab6556	IF: 1:200
chicken anti-Rabbit Alexa Fluor 594	ThermoFisher	a21442	IF: 1:200
chicken anti-Mouse Alexa Fluor 647	ThermoFisher	a21463	IF: 1:200
Goat anti-mouse IgG-HRP	Sigma	AP127P	WB:1: 20000
Goat anti-rabbit IgG-HRP	SCBT	SC-2030	WB: 1:5000
CD56 Pe-Cy7	BD biosciences	557747	FC: 1:100
CD29 APC	Thermofisher	17-0299-42	FC: 1:100

Supplementary Table 1. List of primary and secondary antibodies. Order information and antibody dilutions for immunofluorescence (IF) and western blotting (WB).

mouse

Gene	Forward Primer	Reverse Primer
B2m	TTCTGGTGCTTGTCTCACTGA	CAGTATGTTCGGCTTCCCATTC
Adprhl1	GCCCTCGGCTATGGAAACATC	CTCCCAGGTGAGAGCACAA
Mustn1	GTCTAAGACATACCAGGTCATGC	GCGGCTGAATACAGATGGGG
Nmrk2	GACTTCTTCAAGCCCCAGGAC	AGGAGGAGTACGTGGGTGTC
Cacng7	CGTCACCAAGTTGATCTCTGG	AGACCACCGAGGTCAAGATG
Pla2g7	CTTTTCACTGGCAAGACACATCT	CGACGGGGTACGATCCATTTC
Snx10	AGAGGAGTTCGTGAGTGTCTG	CTTTGGAGTCTTTGCCTCAGC

human

Gene	Forward Primer	Reverse Primer
B2M	GAGGCTATCCAGCGTACTCCA	CGGCAGGCATACTCATCTTTT
DNER	CAGGGACCTCGTTAATGGCT	CGCACTCTTCACCTGTAAACC
ITGB6	TCCATCTGGAGTTGGCGAAAG	TCTGTCTGCCTACACTGAGAG
KCNN3	GCTCCATCACCCTAATGCCA	TGGAGTCCTTTGAGTACAAACCC
MCHR1	CTCACTTCGGCAGGATCACC	TGAAGATGTCGGGGACGTTG
TSPAN15	AGTCCCGGAGAGAACGCC	GCCCCAATCAGCCAGAACAC

Supplementary Table 2. List of primers used for qRT-PCR.
