# Supplementary material for: Three-dimensional tissue-engineered human skeletal muscle model of Pompe disease
Source: Commun Biol. 2021 May 5;4:524. doi: 10.1038/s42003-021-02059-4 (PMC8100136; doi:10.1038/s42003-021-02059-4)
Supplement: Supplementary file 2 — Description of Additional Supplementary Files [file 42003_2021_2059_MOESM2_ESM.pdf]

Description of Additional Supplementary Files

File Name: Supplementary Data 1

Description: Contains disease signature identified by KO vs WT RNA-seq with human orthologs
