# Supplementary material for: Three-dimensional tissue-engineered human skeletal muscle model of Pompe disease
Source: Commun Biol. 2021 May 5;4:524. doi: 10.1038/s42003-021-02059-4 (PMC8100136; doi:10.1038/s42003-021-02059-4)
Supplement: Supplementary file 4 — Reporting Summary [file 42003_2021_2059_MOESM4_ESM.pdf]

nature research

Corresponding author(s): *Double-blind peer review submissions: write DBPR and your manuscript number here instead of author names.*

Last updated by author(s): *YYYY-MM-DD*

Reporting Summary

Nature Research wishes to improve the reproducibility of the work that we publish. This form provides structure for consistency and transparency in reporting. For further information on Nature Research policies, see our Editorial Policies and the Editorial Policy Checklist.

Statistics

For all statistical analyses, confirm that the following items are present in the figure legend, table legend, main text, or Methods section.

n/a	Confirmed	
☐	☒	The exact sample size (*n*) for each experimental group/condition, given as a discrete number and unit of measurement
☐	☒	A statement on whether measurements were taken from distinct samples or whether the same sample was measured repeatedly
☐	☒	The statistical test(s) used AND whether they are one- or two-sided<br>*Only common tests should be described solely by name; describe more complex techniques in the Methods section.*
☐	☒	A description of all covariates tested
☐	☒	A description of any assumptions or corrections, such as tests of normality and adjustment for multiple comparisons
☐	☒	A full description of the statistical parameters including central tendency (e.g. means) or other basic estimates (e.g. regression coefficient) AND variation (e.g. standard deviation) or associated estimates of uncertainty (e.g. confidence intervals)
☐	☒	For null hypothesis testing, the test statistic (e.g. *F*, *t*, *r*) with confidence intervals, effect sizes, degrees of freedom and *P* value noted<br>*Give P values as exact values whenever suitable.*
☒	☐	For Bayesian analysis, information on the choice of priors and Markov chain Monte Carlo settings
☒	☐	For hierarchical and complex designs, identification of the appropriate level for tests and full reporting of outcomes
☐	☒	Estimates of effect sizes (e.g. Cohen's *d*, Pearson's *r*), indicating how they were calculated

Our web collection on statistics for biologists contains articles on many of the points above.

Software and code

Policy information about availability of computer code

Data collection	Custom data collection tools were not used.
Data analysis	Open source software was used to analyze the RNA-seq data as described in the Methods. STAR was used for read alignment, featureCounts and HTSeq for generating gene count data, and DESeq2 was used for analyzing differential gene expression. Gene set enrichment analysis was conducted using the GSEA java software.

For manuscripts utilizing custom algorithms or software that are central to the research but not yet described in published literature, software must be made available to editors and reviewers. We strongly encourage code deposition in a community repository (e.g. GitHub). See the Nature Research guidelines for submitting code & software for further information.

Data

Policy information about availability of data

All manuscripts must include a data availability statement. This statement should provide the following information, where applicable:

- Accession codes, unique identifiers, or web links for publicly available datasets
- A list of figures that have associated raw data
- A description of any restrictions on data availability

Data supporting the main figures of theses studies are available on figshare (https://doi.org/10.6084/m9.figshare.14173013.v1), and the data within the supplemental materials are available upon reasonable request. The sequencing data that support the findings of this study have been deposited in Gene Expression Omnibus with the accession codes GSE159062 and GSE156063. Data referenced in this study are available Gene Expression Omnibus GSE57980 and GSE38680.

Field-specific reporting

Please select the one below that is the best fit for your research. If you are not sure, read the appropriate sections before making your selection.

☒ Life sciences ☐ Behavioural & social sciences ☐ Ecological, evolutionary & environmental sciences

For a reference copy of the document with all sections, see nature.com/documents/nr-reporting-summary-flat.pdf

Life sciences study design

All studies must disclose on these points even when the disclosure is negative.

Sample size	Sample size was determined based on the limited availability of healthy and diseased muscle samples. As a result, 3 healthy and 3 IOPD donors are compared throughout the paper experiments. One exception is in Fig 1h-k where significant differences between representative healthy and disease donors were not observed. With the RNA-seq, an additional healthy donor was added to increase power (a total 4 healthy and 3 IOPD). Due to limited availability of additional healthy (2) and LOPD (2) donors, the analyses of these donors are limited to Supp. Fig. 3.
Data exclusions	No data were excluded.
Replication	Replication was successful in 3 healthy and 3 diseased (infantile-onset Pompe donors).
Randomization	Randomization is not relevant to our study design.
Blinding	Blinding was not possible due to the limited availability and sourcing of healthy and diseased muscle samples.

Reporting for specific materials, systems and methods

We require information from authors about some types of materials, experimental systems and methods used in many studies. Here, indicate whether each material, system or method listed is relevant to your study. If you are not sure if a list item applies to your research, read the appropriate section before selecting a response.

Materials & experimental systems

n/a	Involved in the study	
☐	☒	Antibodies
☒	☐	Eukaryotic cell lines
☒	☐	Palaeontology and archaeology
☐	☒	Animals and other organisms
☒	☐	Human research participants
☒	☐	Clinical data
☒	☐	Dual use research of concern

Methods

n/a	Involved in the study	
☒	☐	ChIP-seq
☐	☒	Flow cytometry
☒	☐	MRI-based neuroimaging

Antibodies

Antibodies used	All antibodies used are described in the Supplementary Information
Validation	Antibodies used for Western blot analysis have been previously validated as follows on their respective product pages as well as in the follow research articles: SAA, GAPDH (Khodabukus et al. 2019); p62, LC3, GAA (Nascimbeni et al. 2012); Lamp2 (Madden et al. 2015); CD56 (Roederer et al. 1996); CD29 (Xue et al 2015).

Animals and other organisms

Policy information about studies involving animals; ARRIVE guidelines recommended for reporting animal research

Laboratory animals	As described in the Methods, male WT and GAA-KO mice (C57BL/6 background) were used as treated at 2 months of age. At 6 months of age, the mice were euthanized for biochemical analyses.
Wild animals	Wild animals were not used.
Field-collected samples	Field-collected samples were not used.
Ethics oversight	The study protocol was approved by Duke Institutional Animal Care and Use Committee (IACUC).

Note that full information on the approval of the study protocol must also be provided in the manuscript.

Flow Cytometry

Plots

Confirm that:

- [x] The axis labels state the marker and fluorochrome used (e.g. CD4-FITC).
- [x] The axis scales are clearly visible. Include numbers along axes only for bottom left plot of group (a 'group' is an analysis of identical markers).
- [x] All plots are contour plots with outliers or pseudocolor plots.
- [x] A numerical value for number of cells or percentage (with statistics) is provided.

Methodology

Sample preparation	Samples were isolated from primary muscle biopsies using outgrowth culture and fixed at passage 5 using 4% PFA. Then, CD56-pecy7 was used to assess the expression of C56 and CD29 cells
Instrument	BD FACSCanto II
Software	FlowJo
Cell population abundance	Cells were not sorted. Cell populations were only analyzed.
Gating strategy	The gating strategy is demonstrated in Figure 1b. Here, strongly positive and negative populations were observed based on the distinct presence of peaks.

- [ ] Tick this box to confirm that a figure exemplifying the gating strategy is provided in the Supplementary Information.
